# Anæmic Vomiting

**Published:** 1904-10-15

**Authors:** 


### ANÆMIC VOMITING.

In a clinical lecture at Guy's Hospital, Dr. Beddard[1] described some cases of what he terms anæmic vomiting. The subjects of the affection are chiefly anæmic women, and the symptoms are pain and vomiting after food. It is essential to distinguish between this condition and gastric ulcer, because in the former the anæmia is the primary trouble, and only harm can result from the strict dietetics necessary for the proper treatment of gastric ulcer. There are several points of distinction. In the first place, the subject of anæmic vomiting will complain of as much pain after drinking a glass of water as after eating a beef-steak, the nature of the ingesta making little difference to the degree of pain which is caused. Again, the tenderness over the upper part of the abdomen is not strictly localised, as it is in gastric ulcer, but is equally marked over the entire stomach area; added to which hæmatemesis will have been absent if the case is one of anæmic vomiting. Another point is that any exertion is likely to accentuate the pain in a marked manner. The symptoms in anæmic vomiting are due to dilatation of the heart consequent on the anæmia. The stretching of the ventricles causes associated hyperæsthesia of a certain area of the skin and of the gastric mucous membrane. Hence the tenderness of the abdomen, the vomiting after food, and the increase in severity of the symptoms on exertion. The main indications for treatment are manifest, if this pathological explanation be accepted. They are: absolute rest in bed, full diet, and the administration of iron and arsenic. The results of treatment are to be estimated by the relief of the symptoms and the rapidity with which the heart regains its normal dimensions.

[1] Guy's Hospital Gazette, October 8, 1904.

